# Time-Dependent Effects of Acute Handling on the Brain Monoamine System of the Salmonid *Coregonus maraena*

**DOI:** 10.3389/fnins.2020.591738

**Published:** 2020-12-04

**Authors:** Joan Martorell-Ribera, Marzia Tindara Venuto, Winfried Otten, Ronald M. Brunner, Tom Goldammer, Alexander Rebl, Ulrike Gimsa

**Affiliations:** ^1^Fish Genetics Unit, Institute of Genome Biology, Leibniz Institute for Farm Animal Biology (FBN), Dummerstorf, Germany; ^2^Psychophysiology Unit, Institute of Behavioural Physiology, Leibniz Institute for Farm Animal Biology (FBN), Dummerstorf, Germany; ^3^Glycobiology Group, Institute of Reproductive Biology, Leibniz Institute for Farm Animal Biology (FBN), Dummerstorf, Germany

**Keywords:** catecholamines, marker genes, monoamine receptors, salmonids, serotonin, stress

## Abstract

The immediate stress response involves the activation of the monoaminergic neurotransmitter systems including serotonin, dopamine and noradrenaline in particular areas of the fish brain. We chose maraena whitefish as a stress-sensitive salmonid species to investigate the influence of acute and chronic handling on the neurochemistry of monoamines in the brain. Plasma cortisol was quantified to assess the activation of the stress axis. In addition, we analyzed the expression of 37 genes related to the monoamine system to identify genes that could be used as markers of neurophysiological stress effects. Brain neurochemistry responded to a single handling (1 min netting and chasing) with increased serotonergic activity 3 h post-challenge. This was accompanied by a modulated expression of monoaminergic receptor genes in the hindbrain and a significant increase of plasma cortisol. The initial response was compensated by an increased monoamine synthesis at 24 h post-challenge, combined with the modulated expression of serotonin-receptor genes and plasma cortisol concentrations returning to control levels. After 10 days of repeated handling (1 min per day), we detected a slightly increased noradrenaline synthesis and a down-regulated expression of dopamine-receptor genes without effect on plasma cortisol levels. In conclusion, the changes in serotonergic neurochemistry and selected gene-expression profiles, together with the initial plasma cortisol variation, indicate an acute response and a subsequent recovery phase with signs of habituation after 10 days of daily exposure to handling. Based on the basal expression patterns of particular genes and their significant regulation upon handling conditions, we suggest a group of genes as potential biomarkers that indicate handling stress on the brain monoamine systems.

## Introduction

The immediate stress response involves the activation of the brain monoaminergic systems, including serotonin (5-hydroxytryptamine; 5-HT), DA and NA as major neurotransmitters. Depending on the type of stressor and the duration and intensity of its occurrence (Barton, 2002), these monoamines modulate the neuronal responses in particular areas of the brain, principally the telencephalon, hypothalamus and brain stem (Winberg and Nilsson, 1993; Kaslin and Panula, 2001). In essence, monoamines affect behavior, the formation of memory and the activity of the brain regions that initiate the neuroendocrine stress axes (Feldman et al., 1995; Viltart and Vanbesien-Mailliot, 2007; Lõrincz and Adamantidis, 2017) to reprogram metabolism, immunity, growth and reproduction (Silbergeld, 1974; Hemre and Krogdahl, 1996). This complex response and the associated physiological changes aim to cope with environmental and/or anthropogenic challenges to reinstall homeostasis (Schreck et al., 2016). Handling procedures are a common anthropogenic disturbance in aquaculture. They activate the stress response in various fish species reflected by increased plasma cortisol concentrations (Barton et al., 1987; Barcellos et al., 2011), altered gene expression in the brain in a specific time-course (Krasnov et al., 2005) and elevated monoaminergic activity in particular brain regions (Gesto et al., 2013). The initial stress response of fish is translated into a systemic stress response via the hypothalamus. It directs the endocrine reaction by initiating the “fight-or-flight response” through the BSC axis (Feldman et al., 1995). This triggers the release of the catecholamines adrenaline and noradrenaline from the head kidney (Reid et al., 1998). Subsequently, the initiation of the BSC axis is followed by the activation of the HPI axis, which causes the release of cortisol into the circulation (Wendelaar Bonga, 1997; Schreck et al., 2016). Eventually, the HPI and the BSC induce a series of compensatory physiological processes to direct the body’s metabolic resources to survival needs (Irwin and Cole, 2013; Aerts, 2018).

**FIGURE 1:**
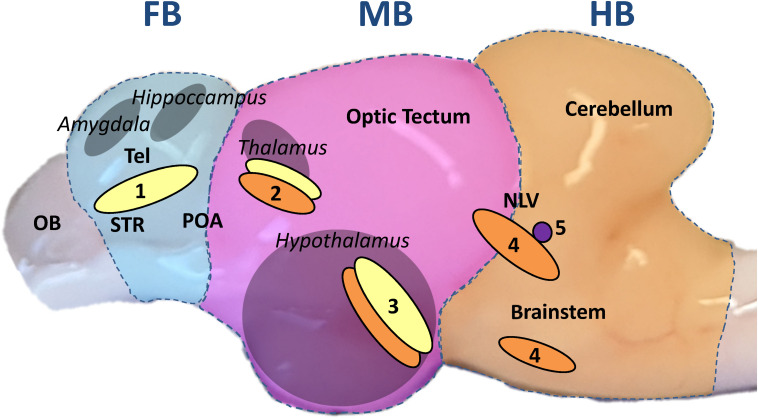
Sagittal view of the whitefish brain. The brain was divided transversally, perpendicular to the rostral-caudal axis, in three parts throughout the experiment: forebrain section (FB, blue underlay); midbrain section (MB, pink); hindbrain section (HB, orange). The FB comprised the telencephalon (dorsal and ventral). The homologous regions to the mammalian amygdala and hippocampus are situated in the dorsal telencephalon while the ventral telencephalon comprises the equivalent to the *striatum* (STR) and dorsally the preoptic area (POA) as described by Mueller (2012); Vindas et al. (2017); Geng and Peterson (2019). The olfactory bulb (OB) was discarded. The MB included every region between optic tectum and diencephalon with the inferior lobes of the hypothalamus from a dorsal-ventral view. The pretectal complex, where the teleost thalamus has been described, is located in the dorsal part of the section (Kaslin and Panula, 2001; Mueller, 2012). The HB was composed of the cerebellum and the brainstem, including the *nucleus lateralis valvulae* (NLV), which was most probably partially divided between MB and HB during dissection (Gaspar and Lillesaar, 2012). From a transversal view, the anterior edge of the cerebellum marked the sectioning line on the brain stem. The different parts of the limbic system are marked in gray and the names in italics. Based on the aforementioned publications on teleost brain organization, monoaminergic neuron populations are marked in yellow for DA neurons; orange for 5-HT neurons and purple for NA neurons: (1) telencephalic complex; (2) pretectal complex; (3) hypothalamic paraventricular nucleus; (4) anterior *raphe nuclei*; (5) *locus coeruleus*.

Monoaminergic neurons project into wide areas of the brain and in particular to those regions that form the limbic system, which evaluates sensory inputs such as visual or olfactory cues as possible threats (Vuilleumier, 2005) and initiates the central stress response with monoamines as active messengers (Morgane et al., 2005). Important monoaminergic neuron clusters are situated in the fish hypothalamus, a control center of the limbic system (Kaslin and Panula, 2001). In mammals, the limbic system is composed of the hypothalamus, hippocampus, amygdala and thalamus, while it is spread between the telencephalon and the midbrain of teleost fishes (Winberg and Nilsson, 1993; Panula et al., 2010; Mueller, 2012) (Figure 1).

Upon synaptic release, monoamines bind to their respective receptors on the target cell. Depending on the type of receptor, this binding can activate or inhibit neuronal functions (Flügge, 1999; Nichols and Nichols, 2008; Mishra et al., 2018). In mammals, several types of receptors for 5-HT (5-HT-1-7), dopamine (D1-5), adrenaline and NA (α1, α2 and β) have been identified (Flügge, 1999; Nichols and Nichols, 2008; Maximino and Herculano, 2010; Fröhlich, 2016; Mishra et al., 2018). However, there is little information on their orthologs in fish. For instance, the adrenergic receptor α2d is not present in mammals, but it is strongly expressed in salmonids (Martorell-Ribera et al., 2020).

In the fish brain, monoamines play an important role in the mechanisms of stress coping. In particular, 5-HT induces different activity patterns in proactive and reactive salmonids (Winberg et al., 1992; Øverli et al., 1999, 2001). In addition, the genes encoding 5-HT receptors (*HTR1A*α and β) in the telencephalon of rainbow trout *Oncorhynchus mykiss* are down-regulated by stress (Moltesen et al., 2016). Such a downregulation of 5-HT receptors may reduce 5-HT uptake and lead to increased 5-HT-metabolism (Dwarkasing et al., 2016). In line with this, the chasing of rainbow trout increased the concentrations of 5-HIAA (the main 5-HT metabolite) in the telencephalon and hypothalamus (Gesto et al., 2013). DA has also an important role in modulating behavioral responses. For example, increased dopaminergic activity in the telencephalon of fish has been linked to avoidance behavior (Höglund et al., 2005) and reward (Teles et al., 2013). The expression of the dopamine receptor 2 (DRD2) gene was up-regulated in the brain of bold zebrafish *Danio rerio* compared to reactive individuals (Thörnqvist et al., 2019). Furthermore, handling and treatment with anesthetics increased the concentrations of DA metabolites in the telencephalon of Arctic charr *Salvelinus alpinus* (Backström et al., 2017) comparable with the aforementioned 5-HT levels in stressed rainbow trout. Also NA, which contributes to vigilance and arousal (Singh et al., 2015), has been subject to increased turnover in rainbow trout under stress conditions (Øverli et al., 2001). In Arctic charr, agonistic interactions increased NA concentrations in the telencephalon of dominant individuals (Backström et al., 2015). Taken together, the observed increase in monoamine metabolites is an indicator for the stress response in different salmonid species.

Maraena whitefish *Coregonus maraena* (Bloch) is a salmonid fish present in the Baltic region in anadromous and landlocked populations (Kottelat and Freyhof, 2007). In Germany, it has been reared for intensive aquaculture production since 2005 (Jansen et al., 2008). Our previous studies revealed that maraena whitefish is highly sensitive to stressors compared to salmonids that are better adapted to husbandry conditions, such as rainbow trout or Atlantic salmon *Salmo salar* (Altmann et al., 2016; Korytář et al., 2016; Rebl et al., 2018). This study investigates the influence of acute and repeated (chronic) handling stress on the monoaminergic systems in different brain regions of maraena whitefish. We focused our research on stress-related changes in plasma cortisol concentrations, monoamine neurotransmitters and metabolites and the expression profile of monoamine-related genes in the brain. Our specific aims were to elucidate (i) which brain regions were activated during handling; (ii) which monoaminergic systems showed increased sensitivity to this type of stressor; (iii) how handling can affect HPI activation; (iv) whether a single episode of handling was quickly overcome or whether it can have long-lasting effects on the monoamine neurochemistry of maraena whitefish and (v) how the monoaminergic system responded over time to repeated episodes of handling. Furthermore, we sought to identify genes that might be suited as animal-based biomarkers for indicating stress in different regions of the brain.

## Materials and Methods

### Husbandry of Maraena Whitefish

The Institute for Fisheries of the State Research Center for Agriculture and Fishery Mecklenburg-Western Pomerania (Born, Germany) and BiMES - Binnenfischerei GmbH (Friedrichsruhe, Germany) provided maraena whitefish for this study. Fish were reared in fresh-water recirculation systems with a stocking density of 10 kg/m^3^ at 18°C and a 12:12-h day-night cycle. Water quality was maintained by automated purification and disinfection (bio-filter and UV light). The concentrations of NH_4_^+^, NO_2_^–^, NO_3_^–^, and NH_3_ in the water, pH, temperature and oxygen saturation were constantly recorded. Commercial dry pellets (4.5 mm; Biomar, Inicio Plus, Aarhus, Denmark) were distributed by automatic feeders at a daily rate of 0.8–4.0% of the biomass of maraena whitefish in the tank.

### Acute and Chronic Handling Experiments

All procedures have been approved by the Landesamt für Landwirtschaft, Lebensmittelsicherheit und Fischerei, Mecklenburg-Vorpommern, Germany (LALLF M-V/TSD/7221.3-1-069/18). The fish (*n* = 48 in total) used in the experiments were juvenile with a starting size of about 20 cm. They were allowed to acclimatize to the recirculation system for at least 2 weeks in the reservoir tank (500 l) and were transferred to the experimental tanks 7 days before the experiments started. These tanks were identical dark polyethylene cylinders with a capacity of 150 L. Experiments were performed in the morning (between 8 and 11 a.m.) to minimize the influence of circadian rhythms on the measurements.

**FIGURE 2:**
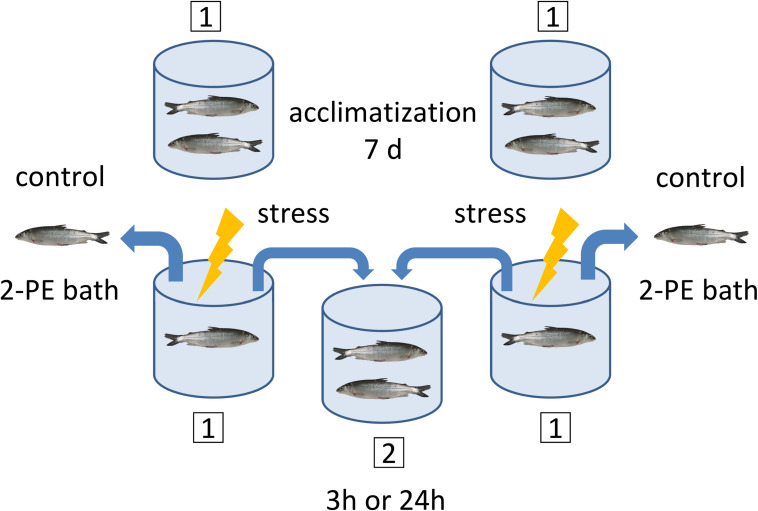
Sampling and handling protocol for the acute experiments. A pair of maraena whitefish was placed in each experimental tank (1) and left undisturbed for acclimatization during 7 days prior to sampling. At day 7, the control fish were sampled for the 3 h or 24 h experiments before stress was applied to the test fish. The timing was as follows: The control fish were caught and placed in a 2-phenoxyethanol (2-PE) bath for anesthetic overdosage, which takes approximately 2–5 min. While the control fish were under anesthesia, the test fish remaining in the tank were subjected to the handling procedure treatment. The test fish were then transferred to the secondary tank (2), where they remained either 3 h or 24 h until sampling. This procedure was applied to two primary tanks simultaneously, where at the end two control fish were in the 2-PE bath and two test fish were transferred to the same secondary tank. The maraena whitefish were always in pairs of two, except for the handling procedure (1 min), during which they were alone in the tank.

Each of the acute handling experiments started with eight rearing tanks with one pair of fish per tank, and four identical secondary tanks without fish (Figure 2). The fish were placed in the tanks in pairs to ensure that the sampling and treatment procedures disturbed the other animals that were part of the experiment as little as possible. At the start of the experiment and before the handling procedure, one fish per pair was euthanized. This fish was designated the control fish (0 h). After sampling of the control fish, the handling protocol was applied for 1 min to the remaining fish, which was designated as test fish. The handling protocol consisted of hunting and catching fish with nets, intermittently lifting the fish out of the water and finally transferring them to a secondary tank, where test fish pairs were left undisturbed for either 3 h or 24 h. After this period, test fish were euthanized and tissues samples were taken as described below (see section “Sampling of Tissues”).

For the chronic handling experiment, control fish (*n* = 8) and test fish (*n* = 8) were kept in pairs in the same condition in eight separate tanks for the duration of the experiment. The experiment was divided into two rounds of eight fish each to swap the tanks for the test fish with the tanks for the controls. The test fish were hunted and netted once a day for 1 min for 10 days, while the controls were left undisturbed. The net lifting and tank-transferring steps performed in the acute-handling procedure were skipped in the chronic handling experiment to avoid skin injuries. To minimize habituation, handling was always performed at a random time within the 12 h ‘daylight’ period. The fish were euthanized 10 days after the onset of the chronic handling procedures, 24 h after the last handling procedure.

### Sampling of Tissues

Before tissue sampling, fish were euthanized with an overdose of 2-phenoxyethanol (0.7 ml/l) followed by spine dissection at the skull level. Sampling and killing methods followed the standards described in the German Animal Welfare Act [§4(3) TierSchG]. Immediately after killing, blood was drawn from the caudal vein using a heparinized syringe and centrifuged at 270 × *g* for 15 min at 6°C to obtain plasma, which was stored at −80°C. Subsequently, the fish brain was quickly and carefully dissected in three parts transversally and perpendicular to the rostral-caudal axis: forebrain section (FB), midbrain section (MB), and hindbrain section (HB). Figure 1 describes in detail the cutting lines of the dissection and the brain parts used in this study. The FB included the telencephalon (dorsal and ventral), while the olfactory bulb was discarded. From the dorsal-ventral view, the MB included each region between the optic tectum and the inferior lobes of the hypothalamus. The HB was composed of the cerebellum and the brainstem. In a transverse view, the anterior edge of the cerebellum marked the line of incision at the brainstem, thereby excluding the spinal cord. The three different sections were separately weighed and homogenized in 500 μl ice-cold PBS. The resulting homogenate was divided into two aliquots and snap-frozen in liquid nitrogen for RNA isolation or monoamine analysis (see sections “Quantification of Monoamines and Metabolites” and “RNA Isolation and cDNA Synthesis”).

### Measurement of Plasma Cortisol

The cortisol concentration in plasma was quantified by a competitive enzyme-linked immunosorbent assay (ELISA; Cusabio Technology, Houston, TX, United States) according to the manufacturer’s instructions. The level of absorbance was measured at 450 nm in a Beckman Coulter DTX 800/880 Series Multimode Detector (Beckman Coulter, Brea, CA, United States).

### Quantification of Monoamines and Metabolites

Concentrations of NA, DA, 5-HT and their metabolites HVA, DOPAC and 5-HIAA were determined in the three brain regions using HPLC with electrochemical detection. This method has been described previously (Otten et al., 2010) and was used with a slightly adjusted extraction procedure. A 250-μl amount of brain samples homogenized in PBS was mixed on ice with 25 μl of 2 M and 25 μl of 0.2 M perchloric acid using a manual homogenizer. Following centrifugation (2,400 × *g*; 4°C; 10 min), the supernatant was preserved on ice while the pellet was resuspended in 300 μl of 0.2 M perchloric acid and centrifuged. The pooled supernatants were mixed and then centrifuged at 37,000 × *g* for 10 min at 4°C. The remaining pellet was weighed with a precision balance and the values obtained were used to calculate the concentration of neurotransmitters in each brain section. Aliquots of 40 μl were analyzed in duplicate. The HPLC system was equipped with a 125 mm × 4 mm reverse-phase column packed with Prontosil C18 AQ (Bischoff Analysentechnik, Leonberg, Germany). The mobile phase consisted of 58 mM sodium hydrogen phosphate buffer containing 1.2 mM octanesulfonic acid, 0.3 mM EDTA, 0.2 mM potassium chloride, and 9% methanol at pH 3.6, and was used at a flow rate of 1.2 ml/min. Electrochemical detection was achieved by a SenCell with a glassy carbon working electrode set at a potential of 600 mV (Axel Semrau GmbH, Sprockhövel, Germany). The HVA/DA, DOPAC/DA and 5-HIAA/5-HT ratios were calculated as an index of DA and 5-HT turnover.

### RNA Isolation and cDNA Synthesis

Total RNA from previously homogenized brain samples was extracted using TRIzol Reagent (Life Technologies). RNeasy Mini Kit (Qiagen, Hilden, Germany) was used to purify the extracted product. The quality of the RNA was analyzed by horizontal electrophoresis on 1%-agarose gels, which validated the presence of intact 18S and 28S rRNA bands for the individual RNA specimens. The RNA concentration was determined with a NanoDrop One^C^ spectrophotometer (Thermo Fisher Scientific, Waltham, MA, United States). The RNA obtained was reverse-transcribed to single-strand cDNA using the SensiFAST cDNA Synthesis Kit (Bioline, London, United Kingdom). The reverse-transcriptase reaction was set at 42°C for 50 min with a subsequent inactivation step at 70°C for 15 min. Finally, the synthetized cDNA was diluted in 80 μl distilled water.

### Real-Time Quantitative PCR (qPCR)

RNA samples from the brains of maraena whitefish exposed to the acute (sampled after 3 h or 24 h) and chronic handling experiments along with the respective controls were analyzed with multiplex qPCR technology (BioMark, Fluidigm, South San Francisco, CA, United States) to study the effects of handling on the transcript level. We designed a primer panel specific for the monoaminergic and neurological stress response. In this matter, orthologous gene sequences from Atlantic salmon *S. salar*, Coho salmon *Oncorhynchus kisutch*, and rainbow trout *O. mykiss* were aligned to the RNA-seq read collection of maraena whitefish *C. maraena* (Brietzke et al., 2016) using the program Bowtie2 (v 2.2.4). The resulting matching alignment with *C. maraena* was sorted and indexed with the Samtools package (v 1.6). Finally, the consensus sequences were visualized and redeemed using the software Ugene (v1.29). These sequences were used to design gene and species-specific primer pairs (see Supplementary Table 1) using the PSQ Assay Design Software 1.0.6 (Biotage AB, Uppsala, Sweden) for amplifying products with final lengths ranging from 140 to 180 bp. In addition, qPCR assays were carried out on 48.48-Dynamic Array IFC chips (Fluidigm) with EvaGreen fluorescence dyes (Bio-Rad, Hercules, CA, United States) using the BioMark HD-System (Fluidigm). One microliter of total RNA was reverse-transcribed using the Reverse Transcription Master Mix (Fluidigm). The resulting cDNA was adjusted to 10 ng/5 μl and underwent a subsequent pre-amplification of 11 cycles performed with the PreAmp Master Mix (Fluidigm) and the primers at a final concentration of 100 μM per primer pair. Afterward, preamplified cDNA was treated with exonuclease I (ExoI; New England BioLabs, Ipswich, MA, United States). Then, the cDNA samples were diluted in SsoFast EvaGreen Supermix with Low ROX (Bio-Rad) and the 20 × DNA Binding Dye Sample Loading Reagent. The cDNA samples and primer-pair mixes were transferred to the corresponding sample and assay inlets of the IFC chip using the IFC Controller RX (Fluidigm) and the ‘Load Mix 48.48 GE’ pre-set script. The IFC chip was transferred to the BioMark HD-System (Fluidigm) to perform the quantification reactions following the ‘GE 48 × 48 Fast PCR + Melt v2.pcl’ cycling program. The obtained qPCR data were analyzed using the Fluidigm RealTime PCR Analysis Software v.4.5.2. All qPCR products were run on a 2%-agarose gel to assess the integrity and specificity of the PCR products. An external standard was used to calculate the relative gene expression, which was normalized against the geometric mean of the copy numbers of *EEF1A1b*, *RPL9* and *RPL32* (Altmann et al., 2015). The 37 genes analyzed belonged to seven gene groups (Table 1). We studied a broad repertoire of genes involved in the synthesis (TH, TPH1, and TPH2) and degradation (MAO) of monoamines, and genes coding for monoamine receptors (ADR, DRD, and HTR) and markers for neuronal activity (FOSL) and neuronal plasticity (BDNF), downstream factors of the monoamine activation (Benito and Barco, 2015; Vindas et al., 2018). Cortisol receptor genes (GR1, GR2, and MR) were analyzed as feedback regulators of the monoamine system (Medeiros and McDonald, 2013). The expression of microglial cell markers (CSF1R and MPEG) was investigated as an indicator for stress effects on this immune cell population (Preston et al., 2018; Kuil et al., 2020).

**TABLE 1:** Target genes.

	**Official gene name; product**	**Function**
Adrenergic receptors	ADRA1B; adrenoceptor α1B	Neuron signaling, regulation of transcription
	ADRA1D; adrenoceptor α1D	Neuron signaling, regulation of transcription
	ADRA2A; adrenoceptor α2A	Neuron signaling, regulation of transcription
	ADRA2B; adrenoceptor α2B	Neuron signaling, regulation of transcription
	ADRA2C; adrenoceptor α2C	Neuron signaling, regulation of transcription
	ADRA2D; adrenoceptor α2D	Neuron signaling, regulation of transcription
	ADRB2; adrenoceptor β2	Neuron signaling, regulation of transcription
	ADRB3A; adrenoceptor β3	Neuron signaling, regulation of transcription
Dopamine receptors	DRD1; dopamine receptor D1	Neuron signaling, regulation of transcription
	DRD2; dopamine receptor D2	Neuron signaling, regulation of transcription
	DRD3; dopamine receptor D3	Neuron signaling, regulation of transcription
	DRD4; dopamine receptor D4	Neuron signaling, regulation of transcription
	DRD5; dopamine receptor D5	Neuron signaling, regulation of transcription
5-HT receptors	HTR1A; 5-hydroxytryptamine receptor 1A	Neuron signaling, regulation of transcription
	HTR1B; 5-hydroxytryptamine receptor 1B	Neuron signaling, regulation of transcription
	HTR1D; 5-hydroxytryptamine receptor 1D	Neuron signaling, regulation of transcription
	HTR1E; 5-hydroxytryptamine receptor 1E	Neuron signaling, regulation of transcription
	HTR1F; 5-hydroxytryptamine receptor 1F	Neuron signaling, regulation of transcription
	HTR2A; 5-hydroxytryptamine receptor 2A	Neuron signaling, regulation of transcription
	HTR2B; 5-hydroxytryptamine receptor 2B	Neuron signaling, regulation of transcription
	HTR2C; 5-hydroxytryptamine receptor 2C	Neuron signaling, regulation of transcription
	HTR3A; 5-hydroxytryptamine receptor 3A	Neuron signaling
	HTR3C; 5-hydroxytryptamine receptor 3C	Neuron signaling
	HTR4; 5-hydroxytryptamine receptor 4	Neuron signaling, regulation of transcription
	HTR6; 5-hydroxytryptamine receptor 6	Neuron signaling, regulation of transcription
	HTR7; 5-hydroxytryptamine receptor 7	Neuron signaling, regulation of transcription
Monoamine synthesis and degradation	TPH1; tryptophan hydroxylase 1	Synthesis of 5-HT
	TPH2; tryptophan hydroxylase 2	Synthesis of 5-HT
	TH; tyrosine hydroxylase	Synthesis of catecholamines
	MAO; monoamine oxidase	Monoamine degradation
Neuronal activity	BDNF; brain derived neurotrophic factor	Growth factor, neurogenesis
	FOSL1; FOS Like 1, AP-1 transcription factor subunit	Transcription factor, neuronal activity
Microglia cell markers	CSF1R (MCSFR); Colony-stimulating-factor-1 receptor	Glia marker, cell differentiation
	MPEG1; Macrophage-expressed gene 1 protein	Glia marker, microbicidal activity
Cortisol receptors	NRC1a (GR1); glucocorticoid receptor 1	Regulation of transcription
	NRC1b (GR2); glucocorticoid receptor 2	Regulation of transcription
	NRC2 (MR); mineralocorticoid receptor	Regulation of transcription

*Summary of the gene groups analyzed for differential expression in the brain of maraena whitefish. Official name, protein product and brief description of the function are indicated.*

### Statistical Analysis

The effects of acute handling (fish sampled 3 and 24 h later) on the neurotransmitter concentration in the brain and cortisol in plasma were analyzed separately for statistical significance using one-way ANOVA and Dunnett’s test for multiple comparisons. In this case, measurements of control groups from the 3 and 24 h experiments (*n* = 16 in total) were compared with the measurements of the fish at 3 h (*n* = 8) or 24 h (*n* = 8) after treatment. The control groups were also used to determine the basal level of neurotransmitters in the brain, which were analyzed using ANOVA followed by Tukey tests. For the 10-day chronic handling experiment, Student’s *t*-test was used to compare the measurements of the control group (*n* = 8) with those of the treated group (*n* = 8) regarding neurotransmitter concentrations in the brain or cortisol levels in plasma. For the gene-expression analysis of the brain, *t*-test was used to compare the transcript level of each gene in the 3- and 24 h- acute-handling groups (*n* = 6 each) and the chronic-handling group (*n* = 6) with their corresponding control groups (*n* = 6 each). We reduced the sample size for gene expression to six in each group to fit all groups onto the same Fluidigm chip for better comparability. The control groups from the acute handling experiment (*n* = 12) were used to determine the gene-expression profile of undisturbed fish between the different brain sections and within the gene groups. These were analyzed using the t-test. Tests were conducted using the software GraphPad Prism 8.0. We defined genes as being significantly expressed if they were up (>2-fold) or down-regulated (<−2-fold) with a *p*-value < 0.05.

## Results

### Effects of Acute and Repeated Handling on Plasma Cortisol Concentration

The concentration of cortisol in plasma was analyzed for the acute (3 and 24 h post-challenge) and repeated handling experiments. In the acute handling experiment, plasma cortisol was significantly elevated (14.2 ± 3.1 ng/ml; *p* < 0.05) 3 h after handling compared to plasma concentrations in control fish (5.4 ± 0.7 ng/ml). At 24 h post-challenge, plasma cortisol (5.5 ± 0.8 ng/ml) did not differ significantly from control levels. After 10 days repeated handling, the cortisol concentration for the challenged fish (7.6 ± 2.3 ng/ml) showed no significant increase over the control fish (4.4 ± 0.5 ng/ml).

### Basal Concentrations of Neurotransmitters and Acute-Handling Effects

**FIGURE 3:**
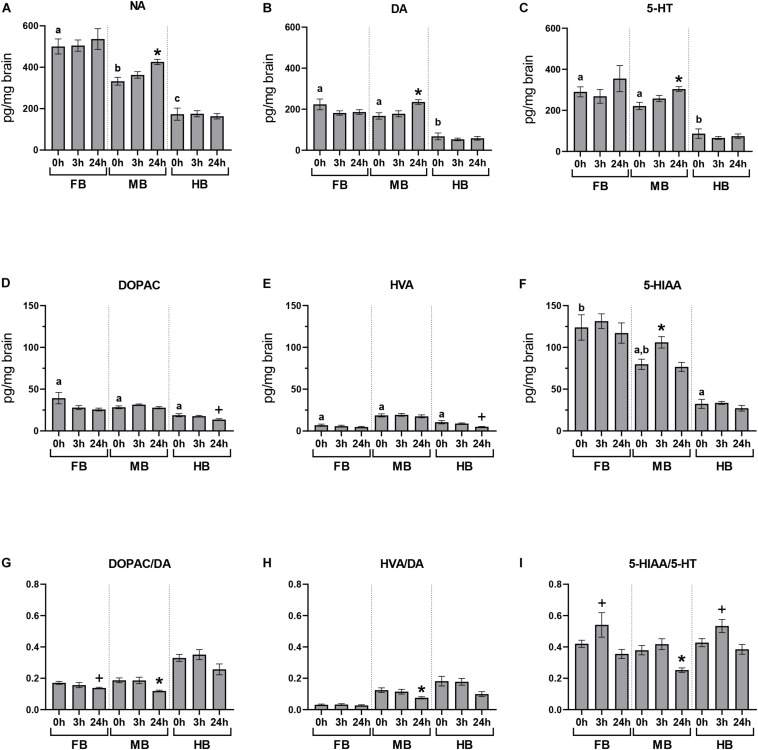
Acute handling effects on neurotransmitter concentrations in the brain of maraena whitefish. The effects of acute stress were analyzed section-wise at 0 h (*n* = 16), 3 h (*n* = 8), and 24 h (*n* = 8) after exposure to acute handling for **(A)** NA; **(B)** DA; **(C)** 5-HT; **(D)** DOPAC; **(E)** HVA; **(F)** 5-HIAA concentrations (pg/mg tissue); and the ratios of **(G)** DOPAC/DA; **(H)** HVA/DA; and **(I)** HIAA/5-HT. Comparisons between handling and control groups were tested for statistical significance using one-way ANOVA and Dunnett’s test for multiple comparisons and is indicated by (*) at *p* < 0.05 or non-significant tendency indicated as (+) at 0.05 < *p* < 0.10. The controls of the acute stress experiments (0 h; *n* = 16) were used to determine the abundance and distribution of neurotransmitters and metabolites in the three brain sections of undisturbed maraena whitefish. Statistical analysis was performed using ANOVA with the Tukey test (*p* < 0.05). Error bars indicate SEM; different characters **(a–c)** above the bars indicate statistically significant concentration differences between brain regions and within time points.

We quantified the basal neurotransmitter concentrations in the different brain regions of undisturbed maraena whitefish (0 h; Figures 3A–F). The highest basal concentrations of neurotransmitters were found in the FB and MB while lowest were in the HB. The DA metabolites DOPAC and HVA were similarly distributed across the three brain regions at low concentrations (Figures 3D,E). The concentration of 5-HIAA showed a rostral-caudal gradient (Figure 3F).

The concentrations of monoamines and their metabolites in the different brain regions were measured at different times after a single episode of acute handling and compared to the baseline concentration of undisturbed fish (Figure 3). Brain neurochemistry was mainly affected by acute handling in the MB. The level of 5-HIAA significantly increased at 3 h post-handling in the MB compared to the undisturbed fish (Figure 3F). The three monoamine neurotransmitters NA, DA, and 5-HT significantly increased in the MB at 24 h after exposure to handling (Figures 3A–C). As the concentrations of metabolites DOPAC, HVA, 5-HIAA did not change (Figures 3D–F), the DOPAC/DA, HVA/DA, and 5-HIAA/5-HT ratios significantly decreased 24 h post-handling in the MB (Figures 3G–I) compared to the control group.

### Expression Profiles of 37 Genes in Different Brain Regions, as Well as Acute-Handling Effects

**FIGURE 4:**
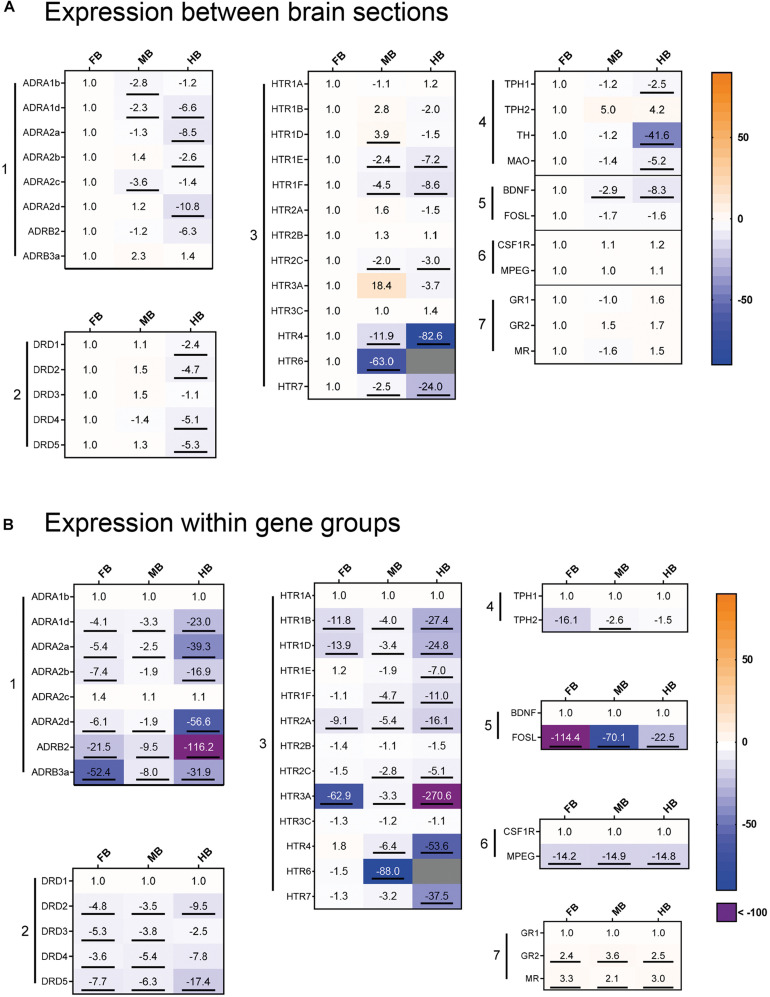
Gene expression profile of maraena whitefish brain. The gene expression was profiled in the control animals of the acute stress experiment (3 and 24 h; *n* = 12) and analyzed with regard to **(A)** the different brain sections and **(B)** to the gene groups expressed. Results are expressed as fold-change values and colored according to the legend on the right. **(A)** To assess the gene expression in the different sections, the gene-specific transcript numbers in the MB and HB are shown relative to the respective transcript levels in the FB, which were set at 1.0. **(B)** Within the gene groups, the transcript numbers of the different genes are shown relative to the respective transcript levels of the gene in the first row, which were set at 1.0. The gene groups comprised (1) adrenergic receptor genes; (2) dopamine receptor genes; (3) 5-HT receptor genes; (4) monoamine synthesis genes; (5) neuronal activity markers; (6) microglia cell markers; (7) cortisol receptor genes. Genes were considered significantly regulated (marked by an underline) in the event of at least a twofold up or down-regulation of the transcript concentration and a *p*-value < 0.05 (Student’s *t*-test).

We profiled the expression of 37 target and three reference genes (Figure 4) across the selected brain regions (Figure 4A) and compared the transcript levels according to their functional categories (Figure 4B). In the FB, the *ADR* and *HTR* genes were most strongly expressed (Figure 4A, gene groups 1 and 3). The 5-HT receptor gene *HTR6* was almost exclusively expressed in the FB and might thus be a suitable marker gene (Figure 4A, gene group 3). Besides this, *HTR4* and *HTR7* were strongly expressed in the FB compared to the MB (−2 to −12-fold) and the HB (−24 to −83-fold; Figure 4A, gene group 3). The *ADRA1d* and *DRD4* transcripts had a decreasing concentration gradient from rostral to caudal brain regions, similar to the 5-HT receptor genes *HTR1E*, -*1F, -2C, HTR4*, and *HTR7*. In contrast, the neurotrophin gene *BDNF* was highly expressed in the FB (Figure 4A, gene group 5), but less expressed in the MB and HB.

In the MB, *ADR*, and *HTR* expression was slightly lower than FB, while *DRD* genes shared similar expression levels in both FB and MB (Figure 4A, gene groups 1–3). *HTR3A* was specifically expressed in the MB and it may be a potential marker gene for this brain region. Similar to *HTR3A*, *HTR1D* was slightly stronger expressed in the MB than in the other brain regions. In contrast, there was less expression of the *ADRA1b* and *ADRA2c* genes in the MB (Figure 3A, gene group 1). The expression of *ADR* and *DRD* genes was markedly lower in the HB than in the other sections, especially for *ADRA2a* and *ADRA2d* (Figure 4A, gene group 1). In contrast, *ADRA1b* and *ADRA2c* were strongly expressed in the whole brain of maraena whitefish, while the *ADRB* genes were least strongly expressed (Figure 4B, gene group 1). Among dopaminergic receptor genes, *DRD1* was most strongly expressed in the brain compared to the other *DRD* genes, which shared similar (though lower) transcript levels (Figure 4B, gene group 2). Among the serotonergic receptor genes, *HTR1A*, -*2B* and -*3C* were the most widely expressed throughout the brain with no differences between brain sections (Figure 4B, gene group 3).

The microglial markers *CSF1R* and *MPEG* were evenly distributed across the three brain sections (Figure 4A, gene group 6), with *CSF1R* being expressed more than ten times as much as *MPEG* (Figure 4B, gene group 6). In the three brain sections, expression of *GR2* and *MR* was two to three times higher than *GR1* (Figure 4B, gene group 7). Among the genes coding for the tryptophan hydroxylase (Tph) enzymes, *TPH1* was slightly less expressed in HB than in FB, while *TPH2* was more strongly expressed in MB and HB than in FB. Regarding the expression of both *TPH* genes, *TPH1* was more strongly expressed in the FB and MB than *TPH2* (Figures 4A,B, gene group 4).

**FIGURE 5:**
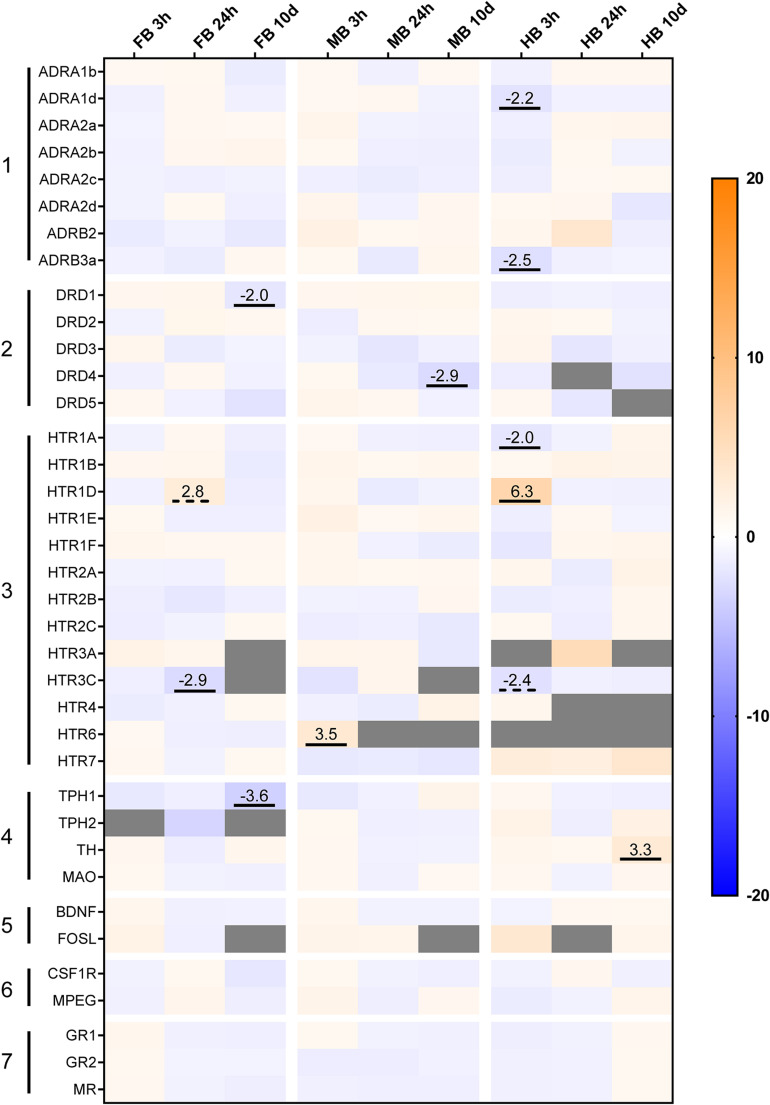
Heatmap illustrating the effects of acute and chronic handling on gene expression in the brain of maraena whitefish. The heatmap shows the gene expression in fold-change values in the different brain sections after acute (3 and 24 h) and chronic (10 days) handling stress. The transcript level of each gene (*n* = 6) in the different brain sections and time-points was compared to its corresponding control group (*n* = 6 each). The scale bar indicates up-regulation (orange) or down-regulation (blue). Gray cells indicate non-detectable expression. The gene groups comprised (1) adrenergic receptor genes; (2) dopamine receptor genes; (3) 5-HT receptor genes; (4) monoamine synthesis genes; (5) neuronal activity markers; (6) microglial markers; (7) cortisol receptor genes. Genes were considered significantly regulated in the event of either an (at least) twofold up- or down-regulation of the transcript concentration and a *p*-value < 0.05 (marked by an underline) or non-significant tendencies at 0.05 < *p* < 0.10 (are marked with dashed lines).

Acute handling affected the expression of genes coding for the adrenergic receptor subtypes *ADRA1* and *ADRB* in the HB region (Figure 5, gene group 1). In particular, *ADRA1d* (−2.2-fold) and *ADRB3* (−2.5-fold) were down-regulated 3 h after handling. Genes coding for the different subtypes of serotonergic receptors were also affected by acute handling throughout the brain (Figure 5, gene group 3). *HTR1D* transcript level increased 6.3-fold after 3 h in the HB and 2.8-fold after 24 h in the FB. *HTR6* was up-regulated (3.5-fold) in the MB 3 h post-handling. *HTR1A* and *HTR3C* were down-regulated (−2-fold) after acute-handling in the HB, while *HTR3C* was also down-regulated (−2.9-fold) in the FB after 24 h.

### Effects of Repeated Handling on Brain Neurotransmitter Concentrations and Gene Expression

**FIGURE 6:**
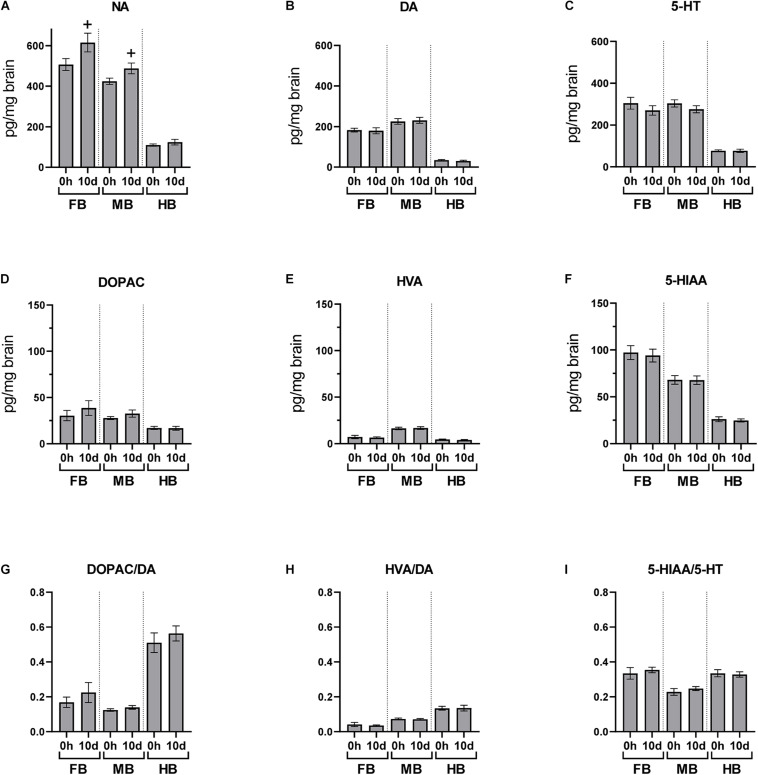
Chronic handling effects on the neurotransmitter concentrations in the brain of maraena whitefish. The effects of repeated handling were analyzed section-wise at 0 h (*n* = 16) and 10 days (*n* = 8) after exposure to acute handling for **(A)** NA; **(B)** DA; **(C)** 5-HT; **(D)** DOPAC; **(E)** HVA; **(F)** HIAA concentrations (pg/mg tissue); and the ratios of **(G)** DOPAC/DA; **(H)** HVA/DA; and **(I)** HIAA/5-HT. Statistical significance was assessed with Student’s *t*-test; non-significant tendency is indicated as (+) at 0.05 < *p* < 0.10.

After the 10-day period of daily handling, brain neurotransmitters were analyzed 24 h after the previous handling episode (Figure 6). NA concentration was slightly increased in FB and MB after 10 days of repeated handling (*p* < 0.10; Figure 6A), but the concentration of the other monoamines and metabolites was unaffected.

The expression of dopaminergic receptor genes was modulated in the FB and MB after 10 days of repeated handling (Figure 5, gene group 2). *DRD1* was −2.0-fold down-regulated in the FB and *DRD4* was −2.9-fold down-regulated in the MB. In addition, the transcript levels of the enzymes involved in monoamine synthesis were modulated in the FB and HB of maraena whitefish. *TPH1* was −3.6-fold down-regulated in the FB, while *TH* was 3.3-fold up-regulated in the HB (Figure 5, gene group 4).

## Discussion

### Low Monoamine Activity in the Hindbrain and Specific Gene Expression Patterns in Brain Sections of Maraena Whitefish

The first part of this study investigated the gene expression and neurochemistry of sixteen individual maraena whitefish, which served as reference individuals in the present study. Although this cohort was considered as “control fish,” it was exposed to unavoidable challenges, i.e., husbandry in the anthropogenic environment as well as the sampling procedure.

**FIGURE 7:**
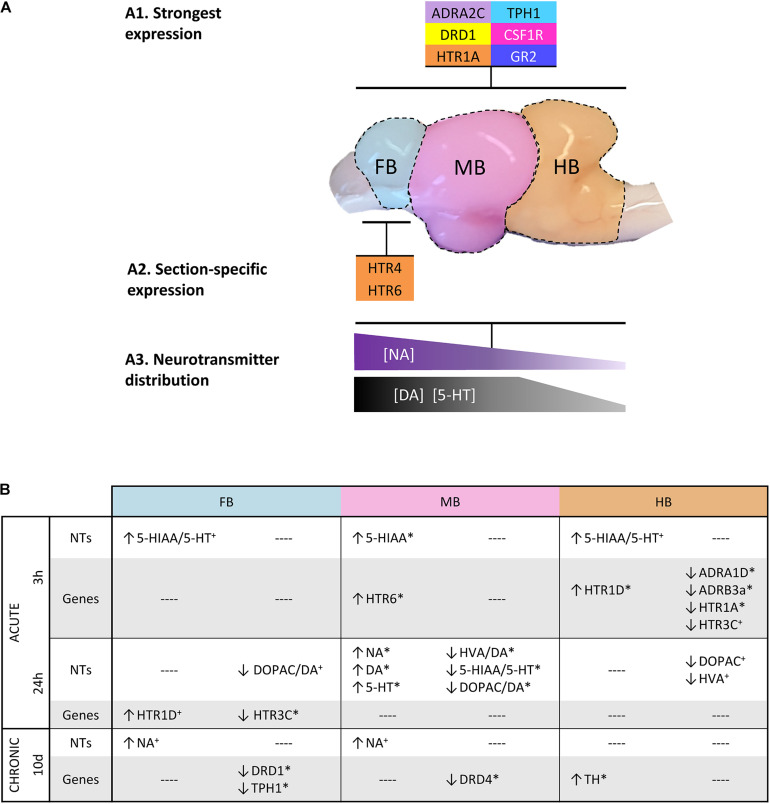
Graphical summary about **(A1)** the most strongly expressed genes with a similar distribution across the three brain sections; **(A2)** those genes that were particularly high expressed in specific sections; and **(A3)** the spatial distribution of specific neurotransmitters. **(B)** Overview of the parameters that were affected by acute and chronic handling at the different time points in the analyzed brain sections. (↑) increased or (↓) decreased ratios/concentrations in case of neurotransmitters (NT) and >2-fold up- or <–2-fold down-regulation in the case of gene expression. Statistical significance is indicated with (^∗^) at *p* < 0.05; non-significant tendency indicated as (+) at 0.05 < *p* < 0.10. See Figure 5 for heatmap with all target genes.

Monoamine concentrations were analyzed in the three selected sections of the brain (Figure 7). We observed a decreasing concentration gradient of the investigated neurotransmitters from FB to HB (Figure 7A). This is consistent with our observation that the genes coding for monoamine receptors are expressed more strongly in FB and MB and less strongly in HB (Figure 4A; gene groups 1–3). This finding may indicate a lower influence of monoamines on HB. Higher concentrations of the monoamines investigated have already been described in the telencephalon and midbrain of the client reef fish *Labroides dimidiatus* and *Naso elegans* (Abreu et al., 2018), rainbow trout *O. mykiss* (Øverli et al., 2001) and Arctic charr *S. alpinus* (Backström et al., 2017). The high basal expression of *ADR* and *HTR* genes in the FB compared to other parts of the brain suggests that the telencephalon is the main target of 5-HT and NA. This assumption was supported by the high level of stored NA in this section, which may indicate the strong innervation of these areas by noradrenergic neurons. The high level of the metabolite 5-HIAA in the FB is most likely the result of 5-HT metabolization processes. The high concentration of monoamines and metabolites in the FB is coinciding with the high expression of *MAO*, which is essential for the degradation of monoamines (Winberg and Nilsson, 1993).

In our study, the expression of *DRD* genes in the HB was generally lower than in the other sections. *DRD1* was the most strongly expressed dopaminergic receptor gene in the three brain sections analyzed in maraena whitefish. In contrast to our findings, *DRD1* of zebrafish *D. rerio* was mainly found in the brain stem and hypothalamic region, but not in the forebrain (Maximino and Herculano, 2010). We found that the expression of *HTR* genes was particularly high in the FB of maraena whitefish compared to the other sections. In humans, prefrontal cortex and hippocampus express almost all *HTR*-encoding genes, which are a main target of serotonergic innervation (Švob Štrac et al., 2016). The corresponding regions of fish are presumably located in the telencephalic area and include the equivalent to the amygdala, POA or the *striatum*, which are involved in the stress response and subjected to 5-HT signaling. Among the HTR genes analyzed, *HTR1A* was the most abundant transcript, which was present in all three brain sections. A similar observation has been made in Gulf toadfish *Opsanus beta* where 5-HT-1A modulates the HPI axis (Medeiros et al., 2010). In mammals, 5-HT-1A is also widely distributed in the brain, mainly present in the limbic areas and the raphe nuclei where it inhibits the 5-HT signaling and modulates the HPA axis (Fuller, 1992; Barnes and Sharp, 1999).

*HTR4* and especially *HTR6* were highly expressed in the telencephalon compared to the rest of the brain (Figure 7A). In mammals, these 5-HT receptors act post-synaptically and induce cAMP signaling upon 5-HT binding (Branchek and Blackburn, 2000; Rosel et al., 2004). A high *HTR4* expression has been observed in progenitors of motor neurons in zebrafish *D. rerio* (Barreiro-Iglesias et al., 2015). In line with our results, mammalian *HTR4* is abundantly present in the mammalian nigrostriatal system that corresponds in teleosts to a region from the diencephalon to the telencephalon (Figure 1) (Barnes and Sharp, 1999; O’Connell et al., 2011). The expression pattern of *HTR6* in the telencephalon of maraena whitefish is similar to that in the brain of the cichlid *Astatotilapia burtoni* (Loveland et al., 2014). Previous studies on zebrafish (Lillesaar, 2011; Gaspar and Lillesaar, 2012) suggest that 5-HT populations express *TPH1* particularly in the hypothalamus (MB in this study), and *TPH2* in the pretectal complex and raphe (MB and HB in this study). Our finding of similar expression levels of *TPH1* in FB and MB of maraena whitefish was unexpected because 5-HT neurons, which normally express this gene, have not been described in telencephalon so far. We could speculate that *TPH1* is expressed by axonal projections of hypothalamic 5-HT populations into telencephalic regions similar to the expression of *TPH2* by neurons from the *raphe nuclei* (Lillesaar et al., 2009). Alternatively, we may have included in the dissection of the FB the pineal gland or part of the posterior *tuberculum*, which have been described to contain *TPH1*-expressing neurons (Gaspar and Lillesaar, 2012).

The LC is situated in the brainstem of teleosts and harbors an important noradrenergic cell population (Ekström et al., 1986). Therefore, the presence of *TH* and the synthesis of catecholamines would be expected here (Kaslin and Panula, 2001). However, *TH* transcripts were almost absent in HB. Concentrations of NA, as well as DA and its metabolites, were also low. The relatively small size of the LC in relation to the HB section might be an explanation for this finding.

Cortisol is the end product of the HPI axis and a strong regulator of the monoaminergic response in the brain (Medeiros and McDonald, 2013). The expression of the cortisol receptor genes *GR2* and *MR* was significantly higher throughout the brain than that of *GR1*, which suggests that these receptors might play a stronger role in cortisol signaling in the brain, compared to other organs where GR1 is predominant (Martorell-Ribera et al., 2020).

The expression of *BDNF*, which is an important factor for neurogenesis and cell proliferation, was particularly high in the FB. The brain of the killifish *Nothobranchius furzeri* displayed a similarly pronounced expression of *BDNF* in the dorsal telencephalic areas, suggesting that this region is essential for neuronal growth and plasticity (D’Angelo et al., 2014).

### Acute Handling Increased 5-HT Turnover and Modulated Receptor Expression at 3 h Post-handling

Brain neurochemistry responded to acute handling with significantly increased 5-HIAA in the MB at 3 h post-handling, together with a slightly increased 5-HT turnover in FB and HB. We speculate that this is the result of an elevated release of 5-HT during and immediately after handling. A previous study demonstrated an increased 5-HT turnover in the telencephalon and hypothalamus of rainbow trout induced by chasing (Gesto et al., 2013). In addition, increased 5-HIAA concentrations have been linked to isolation and confinement stress of rainbow trout (Øverli et al., 2001). In Arctic charr, 5-HT and 5-HIAA concentrations in the brain stem of dominant fish were lower than in subordinate fish (Backström et al., 2015). In our study, serotonergic activation was accompanied by significantly elevated plasma cortisol levels 3 h after treatment, which indicates an activated HPI axis (Wendelaar Bonga, 1997). Although this cortisol concentration was significantly higher than that of control fish, it was still below the established stress levels of rainbow trout (Iwama et al., 1999; Barton, 2000; Schreck et al., 2016). This may be an indication that the cortisol concentration may have been higher before our first sampling, while it was already decreasing at the time of sampling. Acute handling up-regulated the *HTR6* expression in the MB of maraena whitefish. The basal expression of *HTR6* in the MB is extremely low and this condition might indicate its rather subordinate role in the response to handling, especially in view of the high *HTR6* expression in the FB of undisturbed fish.

The HB of maraena whitefish was expected to have important 5-HT neuron populations, which have been found in the NLV and *raphe nuclei* of several teleost species (Lillesaar, 2011). However, lower basal concentrations of 5-HT and 5-HIAA were observed in the HB compared to the other sections. This section displayed weakly increased 5-HT turnover 3 h after handling (Figure 7B). Contrary, acute stressors have been reported to increase of 5-HIAA/5-HT ratios in the brain stem of rainbow trout (Gesto et al., 2008).

Our results showed that the expression of the *HTR1* family genes was affected 3 h after handling. *HTR1D* was up-regulated, while *HTR1A* was downregulated. In mice, 5-HT-1D regulates the activity of 5-HT neurons in the *raphe nuclei* (Vogelgesang et al., 2017) by inhibiting 5-HT actions (Huang and Thathiah, 2015). 5-HT-1A controls the HPA axis in mammals and also inhibits 5-HT signaling and, at the same time, it is regulated by a negative feedback after cortisol release (Fuller, 1992; Zhong and Ciaranello, 1995; Barnes and Sharp, 1999). In rainbow trout, *HTR1A* was down-regulated in the telencephalon 1 to 4 h post-stress (Moltesen et al., 2016), while it was upregulated in subordinate Atlantic salmon parr (Thörnqvist et al., 2015).

5-HT-3C promotes 5-HT signaling in mammals (Sangkuhl et al., 2009). In this study, *HTR3C* was significantly down-regulated in the brain of maraena whitefish 24 h post-handling (Figure 7B). The up-regulation of *HTR1D* and down-regulation of *HTR3C* are most likely compensatory responses to counteract this 5-HT release, which could affect the *raphe* 5-HT populations of the brain stem, as described in brook trout *Salvelinus fontinalis* and other teleosts (Bolliet and Ali, 1992; Lillesaar, 2011). The adrenergic receptor genes *ADRA1D* and *ADRB3a* were also down-regulated in the HB 3 h post-handling. Both adrenergic receptors act post-synaptically as activating modulators of NA actions in the target neuron (Graybiel and Penney, 1999; Huang and Thathiah, 2015; Maletic et al., 2017). *ADRA1D* has been shown to be up-regulated in the hippocampus of rats under restraint stress (Campeau et al., 2010). *ADRB3* genes code for β_3_ receptors, which are generally involved in NA release and neuron plasticity and are down-regulated during chronic stress in mammals (Seki et al., 2018). Although no changes in NA brain levels were observed 3 h post-handling, the down-regulation of these two genes suggests that acute handling might result in desensitization to NA in the HB.

### The Initial Response to Handling Was Compensated by Increased Monoamine Concentrations at 24 h Post-handling

The initial serotonergic response 3 h post-handling was followed after 24 h by increased concentrations of NA, DA and 5-HT in the MB compared to control fish (Figure 7B). We assume that the increased monoamine concentrations are the compensatory response to the stress-related demand of monoamines to cope with stress. The observed increase in neurotransmitters could be explained by a reduced monoamine metabolism. However, the metabolites were not reduced compared to the value at 0 h and the gene expression of *MAO* was unaffected by stress. Alternatively, the elevated monoamine concentrations could be the result of an increased synthesis as a consequence of an accelerated enzymatic activity of TPH and TH or the increased availability of their substrates tryptophan and tyrosine. Both enzymatic activity and substrate availability are likely to be affected by stress as previously shown (Dunn and Welch, 1991; Chen and Miller, 2012). However, we did not detect an increased *TPH* and *TH* gene expression at sampling times of 3 and 24 h. Unfortunately, our data do not permit a final conclusion because we have neither analyzed the protein content expression nor the activity of the relevant enzymes. We think that the unchanged metabolite concentrations and return of plasma cortisol levels to baseline 24 h after the challenge reflect recovery from stress and a return of HPI activity to baseline between 3 and 24 h after the challenge. In teleost fish, low reactivity to stress may indicate an increased allostatic load (Madaro et al., 2015; Moltesen et al., 2016), which also impairs the natural stress-response mechanisms (Schreck et al., 2016). This implies the stress-dependent activation of the DA and NA systems, although we did not detect any significantly altered levels of DA or NA in the brain of maraena whitefish 3 h post-handling. After 24 h post-handling, only the expression of 5-HT-related genes in the FB was affected (Figure 7B). The down-regulation of *HTR3C*, which activates 5-HT signaling, and the up-regulation of *HTR1D*, which antagonizes the 5-HT signaling, suggest a balanced 5-HT activity that migrated from the rear brain at 3 h post-handling to the telencephalon 24 h post-handling.

### Repeated Handling Evoked a Weak Monoaminergic Response

The 10-day repeated handling had no significant effects on the brain neurochemistry (Figure 7B). Although the fish in the chronic experiment were sampled 24 h after the last treatment, the monoaminergic activity was not consistent with the observed recovery period 24 h after acute treatment. Explanations for these observations could be habituation processes, exhaustion of neural responses or the effects of chronically elevated cortisol levels on the monoaminergic systems. The possibility that the decreased neurochemical response could be due to continuously elevated cortisol levels in the brain could be excluded, since the cortisol levels of repeatedly handled fish did not differ from those of control fish at the end of the 10-day experiment and were not significantly elevated even 24 h after acute handling. Moreover, the observed plasma cortisol levels remained below the stress levels described for rainbow trout (Schreck et al., 2016). Increased DA metabolite concentrations without altered plasma cortisol levels have been previously described in Arctic charr in response to handling (Backström et al., 2017). Depletion or exhaustion of neuronal responses are not very likely as we did not observe a reduction of the neurotransmitter pool compared with controls, which might decrease the monoaminergic response. Instead, we found subtle modulations of the *TPH1* and *TH* expression. This suggests that the monoamine synthesis pathways were sensitive to repeated handling and may indicate habituation.

Isolated rainbow trout had increased NA levels in the telencephalon and the optic tectum after 1 week (Øverli et al., 1999, 2001). Our results are consistent with these reports, since we also observed slightly increased concentrations of noradrenaline together with an upregulation of *TH* in HB.

Previous studies on the POA in the brain of cichlid fish revealed increased levels of tyrosine hydroxylase after handling stress, and a modulated expression of dopamine receptors and genes related to 5-HT synthesis after repeated handling (Chabbi and Ganesh, 2015).

*DRD1* and *DRD4* were down-regulated in the FB and MB of maraena whitefish, respectively. D1 activates the dopamine signaling, while D4 inhibits it (Svingos et al., 2000; Centonze et al., 2003; Mizuno et al., 2007). In studies with rodents, acute and chronic stress modulated the expression of *DRD1*, depending on the forebrain region analyzed (Rasheed et al., 2012). The DA receptor D4 participates in the dopaminergic stress response of primates (Arnsten et al., 2000). Taking into account the aforementioned studies, the down-regulation of *DRD1* and *DRD4* in our results points to the desensitization of the DA system in the FB and MB.

Our results suggest that the 5-HT system was desensitized by a repeated stressor compared to the 5-HT response to a single episode of handling (see sections “Acute Handling Increased 5-HT Turnover and Modulated Receptor Expression at 3 h Post-handling” and “The Initial Response to Handling was Compensated by Increased Monoamine Concentrations at 24 h Post-handling”). However, markers derived from the synthesis pathway of 5-HT are apparently promising indicators of the serotonergic response to chronic stressors. TPH controls the rate-limiting step of synthetizing 5-HT from tryptophan. Recently, a study demonstrated a downregulation of *TPH1* and *TPH2* after heat stress in medaka fish *Oryzias latipes* (Shimomura et al., 2019). In our experiment, the repeated handling reduced *TPH1* expression in the FB section of maraena whitefish. However, as discussed in Section “Low Monoamine Activity in the Hindbrain and Specific Gene Expression Patterns in Brain,” the expression of *TPH1* was detected in 5-HT neurons, which should not be present in the brain parts included in our FB sections. Therefore, further investigations based on precise histology are required to confirm our data.

## Conclusion

The effects of stress on the brain neurochemistry vary remarkably throughout the numerous interrelated regions of the brain. In this study, we observed that acute handling activates the HPI axis and serotonergic activity of the brain shortly after the challenge. At 24 h after acute handling, the return of plasma cortisol to baseline together with an increase of brain monoamine concentrations indicated a recovery. After 10 days of repeated handling, the modest neurochemical response and the low cortisol levels might reflect a habituation to the persistent challenge, as the increase of the NA concentration is much weaker than at 24 h after the single handling event. Based on the basal expression and the significant regulation under handling conditions, we selected a group of genes as potential markers that indicated the effects of handling as a stressor on the brain monoamine systems, i.e., *DRD1*, *DRD4*, *ADRA1D*, *ADRB3a*, *HTR1A*, and *HTR3C*. Our results suggest that maraena whitefish should be left undisturbed for at least 24 h following routine aquaculture procedures that include intense handling, such as size sorting or transportation. This salmonid species apparently habituates to repeated handling in the long term. Our study shows that a stress-sensitive species such as maraena whitefish is able to adapt to the anthropogenic stressors related to aquaculture conditions if enough time is made available between challenges.

## Data Availability Statement

Datasets generated for this study are available from the corresponding authors on request.

## Ethics Statement

The animal study was reviewed and approved by Landesamt für Landwirtschaft, Lebensmittelsicherheit und Fischerei Mecklenburg-Vorpommern, Germany (LALLF M-V/TSD/7221.3-1-069/18).

## Author Contributions

UG, AR, JM-R, and TG designed the research. UG, AR, and TG supervised the experiments. RB organized the husbandry of the maraena whitefish. JM-R and MV performed stress experiments and sampled fish. JM-R and WO performed HPLC analyses. JM-R and AR conducted RT-qPCR analyses. JM-R performed statistical analyses. JM-R wrote the manuscript. AR and UG edited the manuscript. All the authors have read and agreed to the final version of the manuscript.

## Conflict of Interest

The authors declare that the research was conducted in the absence of any commercial or financial relationships that could be construed as a potential conflict of interest.

## Supplementary Material

Supplementary Table 1 Target genes and primers designed for RT-qPCR analysis.

## Abbreviations

5-HIAA: 5-hydroxyindoleacetic acid
5-HT: 5-hydroxytryptamine
BSC: brain-sympathetic-chromaffin axis
DA: dopamine
DOPAC: 3,4-dihydroxyphenylacetic acid
FB: forebrain
MB: midbrain
HB: hindbrain
HPA: hypothalamic-pituitary-adrenal axis
HPI: hypothalamic-pituitary-interrenal axis
HPLC: high-pressure liquid chromatography
HVA: homovanillic acid
LC: *locus coeruleus*
NA: noradrenaline
NLV: *nucleus lateralis valvulae*
POA: pre-optic area
STR: *striatum*.
