# Supplementary material for: Time-Dependent Effects of Acute Handling on the Brain Monoamine System of the Salmonid Coregonus maraena
Source: Front Neurosci. 2020 Dec 4;14:591738. doi: 10.3389/fnins.2020.591738 (PMC7746803; doi:10.3389/fnins.2020.591738)
Supplement: Supplementary Table 1 — Target genes and primers designed for RT-qPCR analysis. [file Data_Sheet_1.PDF]

Supplementary Material

Supplement figure 1. List of target genes and corresponding primers designed for RT-qPCR analysis.

Official gene symbols and names	NCBI accession code used for alignment (Bowtie2)	Sense, antisense primer (5'→3')	Function
(1) Adrenergic receptor genes			
ADRA1B; adrenoceptor α1B	NM_001124675	CCGGGAGAAGAAAGCAGCTAAA, AGTTGAAGTAGCCCAGCCAGAA	Neuron signalling, regulation of transcription
ADRA1D; adrenoceptor α1D	NM_001124650	TCCGTGCGCTTGATGAAGTTCT, AGACCATGTCAGATGGCTTCAG	Neuron signalling, regulation of transcription
ADRA2A; adrenoceptor α2A	XM_021559186	TCCGCCCTATAGCCTCCAGAT, GACGCCAACGAAACCAGGAAG	Neuron signalling, regulation of transcription
ADRA2B; adrenoceptor α2B	XM_021621844	AGCCACAGCGCTCTTTGCTAC, GGATGATGAGGGTCGCCACTA	Neuron signalling, regulation of transcription
ADRA2C; adrenoceptor α2C	XM_021595607	TGTGGCTGATCTCGGCTGTTAT, AACCTGGTAGATCCTGATGAAGA	Neuron signalling, regulation of transcription
ADRA2D; adrenoceptor α2D	XM_021619839	GTAGTGGCCGTTTTCACCAGC, CACACCAGGTGCTCCCAAAGT	Neuron signalling, regulation of transcription
ADRB2; adrenoceptor β2	NM_001124440	TTCCAGCGGCTGCAGACGGT, AAGACGTCCGCCGCTGTCCA	Neuron signalling, regulation of transcription
ADRB3A; adrenoceptor β3	NM_001124628	GTTTGGATTGTGTCTGCTCTGAT, TGAGCAAAGGGATGTAGAATGAC	Neuron signalling, regulation of transcription
(2) Dopamine receptor genes			
DRD1; dopamine receptor D1	XM_021617454	CAAGCCCATTCCGCTACGAGA, AGTCGCAGTTGTCCGGAGGC	Neuron signalling, regulation of transcription
DRD2; dopamine receptor D2	NM_001124372	CAAAGTGGCTAAAGCGTTTCAGA, CATATGATAAATACACCCAGGACT	Neuron signalling, regulation of transcription
DRD3; dopamine receptor D3	XM_021620316	TACTCATCCTGGCCATCGTGTT, CTCCGACCACCTCACAGGTAT	Neuron signalling, regulation of transcription

DRD4; dopamine receptor D4	XM_021598851	ATGTGGAGTTCAAGGATGGTCTA, CTTACGGTTGTAGTTCAGTGGG	Neuron signalling, regulation of transcription
DRD5; dopamine receptor D5	XM_021575786	TGGACTGCAACGCAAGCTTGAA, GTGTTCCACGGCTCTCTCCAA	Neuron signalling, regulation of transcription
(3) 5-HT receptor genes			
HTR1A; 5-hydroxytryptamine receptor 1A	XM_021606534	AGTCGTTGGAGATCATAGAAGTTA, TTTACCGTTTTCCTCTCCCTGTA	Neuron signalling, regulation of transcription
HTR1B; 5-hydroxytryptamine receptor 1B	XM_021584469	TCTCATAGCGTCTCTAGCTGTC, GAAGGATAGAAGCAGTGCAACAT	Neuron signalling, regulation of transcription
HTR1D; 5-hydroxytryptamine receptor 1D	XM_021574723	CTCCAGCAAACTTTCTCATAGCA, ACGCATAAGTGAAGGATAGAAGC	Neuron signalling, regulation of transcription
HTR1E; 5-hydroxytryptamine receptor 1E	XM_021611723	GGAGAGGAAGGCAGCACGTAT, TTGATGTAGCCCAGCCAGGTTA	Neuron signalling, regulation of transcription
HTR1F; 5-hydroxytryptamine receptor 1F	XM_021579459	CGTAACCAGGAAGTTGCATCAC, TGTCAACACTCAGCCACATGTAA	Neuron signalling, regulation of transcription
HTR2A; 5-hydroxytryptamine receptor 2A	XM_021597601	TTACGGTGACGGGTAACATCCT, CACCAGTGGTCATAGAGAATTGT	Neuron signalling, regulation of transcription
HTR2B; 5-hydroxytryptamine receptor 2B	XM_020480170	TGTTCTCAGATTCCGGGTGGC, GCTTTGGCCCTGGACTTATACT	Neuron signalling, regulation of transcription
HTR2C; 5-hydroxytryptamine receptor 2C	XM_021577649	TGTTCTCTTCTCTACAGCCAGTA, TAGGCATTGACACTCCTATAGATA	Neuron signalling, regulation of transcription
HTR3A; 5-hydroxytryptamine receptor 3A	XM_021611929	CATCACGTGTCGGACATAAGGA, TTTCTCCCTGATCGATTTCTGATA	Neuron signalling
HTR3C; 5-hydroxytryptamine receptor 3C	XM_021559389	CCCATCATCCGTGAGCACTTC, AGGCCTCTTCATTGTCTGTGTC	Neuron signalling
HTR4; 5-hydroxytryptamine receptor 4	XM_021583604	ACCGCCAGCTCAGGTTAATCAA, AGGCTGTTGTCAACAGTACATCT	Neuron signalling, regulation of transcription
HTR6; 5-hydroxytryptamine receptor 6	XM_021616202	TGGCGGCGCTAACTCACCCT, GTTCACCGACAACGGTACATGA	Neuron signalling, regulation of transcription
HTR7; 5-hydroxytryptamine receptor 7	XM_021621855	TCTCTCCGTGGCAATAGTTGTC, TCCCCAGATACCTGTCAACGC	Neuron signalling, regulation of transcription

(4) Monoamine synthesis and degradation genes			
TH; tyrosine hydroxylase	XM_021564247	GTTCGAGACGTTTGAAGCTAAGA, TTTTGACGTCCTCTGCGATCCT	Synthesis of L-DOPA from L-tyrosine
DBH; dopamine β-hydroxylase	XM_021622665	ATCATAGAGGTCGTAAGGACAAC, GAGGCAGGGCAGTTTGTGTACA	Synthesis of Noradrenaline from Dopamine
PNMT; phenyl-ethanolamine N-methyltransferase	XM_021565302	TTGACCCGGCAGCCTATCTAC, CGCTCAACACCTGGTATAGAGT	Synthesis of adrenaline from noradrenaline
TPH1; tryptophan hydroxylase 1	XM_021598622	AAGGCGTAACTCTGAATTCGAGA, GGGGCATTTTCTATATCTTCTTCA	Synthesis of 5-HTP from L-Tryptophan
TPH2; tryptophan hydroxylase 2	XM_021576444	CAGGCTTGCTGTCATCTATTGG, TTAGCAAACTCCCTCATCTTCTC	Synthesis of 5-HTP from L-Tryptophan
MAO; monoamine oxidase	NM_001124688	CCCTGATGGACTACAACAACCT, TCGCAGAACTGGTCCAGCAGA	Monoamine degradation
(5) Neuronal activation marker genes			
BDNF; brain-derived neurotrophic factor	XM_021607563	AGTTACCGTCCTGGAAAAGGTC, TCGCACGTAGGACTGGGTTGT	Growth factor, neurogenesis,
FOSL1; FOS Like 1, AP-1 transcription factor subunit	XM_021583898	AGCCCTCCCTCATCGGTCCA, CTCTGGGGACAAATGTTCATCG	Transcription factor, neuronal activity
(6) Microglial marker genes			
MPEG1; macrophage-expressed gene 1 protein	XM_021605220	CATCAACACTTGGCTGGAACAG, GTTCCGTACTTGAACACGAGCG	Glia marker, microbicidal activity
CSF1R (MCSFR); Colony-stimulating-factor-1 receptor	NM_001124739	TGCAACGTCCCGGTGAATACAA, ACAGCTACCTGGAGATGAGGC	Glia marker, cell differentiation
(7) Cortisol receptor genes			
NRC1a (GR1); glucocorticoid receptor 1	NM_001124730	CACAGTACCAAAGGATGGATTGA, TGCATGGAGTCCAGTAGCTTAG	Regulation of transcription
NRC1b (GR2); glucocorticoid receptor 2	NM_001124482	TGCCCCTCAGACTTTTACGTAG, TGGTGGTGTTGGAACCGCTAAA	Regulation of transcription
NRC2 (MR); mineralocorticoid receptor	NM_001124483	TGCTCAGCCCAGTTCCCAAAGA, TGCATGGCGTCCAGTAGCTTG	Regulation of transcription
